# Properties of odor identification testing in screening for early-stage Alzheimer’s disease

**DOI:** 10.1038/s41598-023-32878-w

**Published:** 2023-04-13

**Authors:** Egle Audronyte, Gyte Pakulaite-Kazliene, Vaiva Sutnikiene, Gintaras Kaubrys

**Affiliations:** Clinic of Neurology and Neurosurgery, Institute of Clinical Medicine, Faculty of Medicine, Vilnius University, Vilnius, Lithuania

**Keywords:** Alzheimer's disease, Dementia

## Abstract

Odor identification (OI) is impaired in the early stages of Alzheimer’s disease (AD). However, data regarding the diagnostic properties of OI tests are lacking, preventing their clinical use. We aimed to explore OI and determine the accuracy of OI testing in screening for patients with early AD. In total, 30 participants with mild cognitive impairment due to AD (MCI-AD), 30 with mild dementia due to AD (MD-AD), and 30 cognitively normal elderly participants (CN) were enrolled, and cognitive examination (CDR, MMSE, ADAS-Cog 13, and verbal fluency tests) and assessment of OI (Burghart Sniffin’ Sticks odor identification test) were performed. MCI-AD patients scored significantly worse in OI than CN participants, and MD-AD patients had worse OI scores than MCI-AD patients. The ratio of OI to ADAS-Cog 13 score had good diagnostic accuracy in differentiating AD patients from CN participants and in differentiating MCI-AD patients from CN participants. Substitution of ADAS-Cog 13 score with the ratio of OI to ADAS-Cog 13 score in a multinomial regression model improved the classification accuracy, especially of MCI-AD cases. Our results confirmed that OI is impaired during the prodromal stage of AD. OI testing has a good diagnostic quality and can improve the accuracy of screening for early-stage AD.

## Introduction

Dementia is a leading cause of disability and dependency globally^1^. With its increasing prevalence, which is expected to reach 78 million cases by 2030, and consequently increasing socioeconomic impact, dementia is becoming a healthcare priority worldwide^1,2^. Despite this, most patients remain undiagnosed, with Alzheimer’s Disease International (ADI) estimating 75% of undiagnosed dementia cases and the number potentially reaching 90% in some low- and middle-income countries^2^. Therefore, identifying affordable and widely accessible measures to achieve accurate diagnosis is increasingly important.

Alzheimer’s disease (AD) is the most common cause of dementia and is estimated to cause 60–70% of all cases^3^. At present, biomarkers used for diagnosing AD require cerebrospinal fluid analysis or advanced neuroimaging techniques, as blood-based biomarkers are not yet accessible for use in clinical practice worldwide. These biomarkers measure brain amyloid-beta (Aβ) protein deposition and neuronal degeneration^4,5^. However, their wide use is limited because testing for them is invasive and expensive. To select patients who would benefit from these testing methods, identifying markers that would be easily accessible in community settings is necessary for the screening of wide populations.

These screening markers should not only reliably identify patients with AD but should also be able to do so in the early stages of the disease. In 2022, most medications used in clinical trials for AD were disease-modifying therapies (83.2%)^6^. These therapies are predominantly aimed at patients with early-stage AD, and as they enter clinical use, the need for accurate and affordable screening measures becomes more apparent.

Olfactory dysfunction in AD patients has been studied for nearly 50 years at this point^7^. Numerous studies have confirmed that it is a common symptom, present in up to 90% of patients with AD^8,9^. However, some uncertainties remain regarding its prevalence and magnitude during the early stages of the disease. Olfactory impairment has been found not only in patients with mild cognitive impairment (MCI)^10–12^ but also in those with subjective cognitive decline (SCD)^13,14^. However, studies with MCI and SCD patients often use variable definitions of subtypes or do not subtype cognitive impairments at all^11,13^. This complicates the interpretation of the results with regard to AD, as it is likely that a heterogeneous mix of MCI and SCD patients with varying neurological conditions is being analyzed together^11^.

Results from longitudinal studies support the early occurrence of olfactory dysfunction in patients with AD and suggest that olfactory testing could be used to predict future cognitive decline during follow-up. In longitudinal studies, olfactory impairment was found to be associated with an increased risk of MCI in healthy individuals^15–18^ as well as with an increased risk of conversion to dementia in patients with MCI^19–22^. In contrast, intact olfactory abilities were found to be reliable in identifying individuals who rarely transition to dementia in the future^23^. However, longitudinal studies often experience the same limitation of inconsistent subtyping of cognitive impairments. In many instances, MCI has not been typed, whereas in some studies, the cause of dementia is not specified^16,21^. Nevertheless, longitudinal studies confirm the rationale for further research on olfactory testing as a screening marker of AD.

The pathological evidence supports these clinical findings. Structures involved in the processing of olfactory information (especially entorhinal and transentorhinal areas) are affected by AD pathology early in the course of the disease^24,25^. Functional magnetic resonance imaging (fMRI) and [18F]-fluorodeoxyglucose PET (FDG-PET) also confirmed structural and functional abnormalities of olfaction-related regions in AD patients as early as SCD^26–29^.

Thus, previous studies indicate that olfactory testing is a promising method for improving the accuracy of screening for early-stage AD and could be introduced into clinical practice if more data are obtained using generally accepted AD diagnostic criteria and standardized assessment methods.

In the current study, we aimed to analyze odor identification in patients with early-stage AD and explore its diagnostic qualities as a screening measure. We hypothesized that odor identification is impaired in the early stages of the disease and can be reliably used to differentiate patients with AD from cognitively normal participants, even in the prodromal stage of AD.

## Results

### Demographic and clinical characteristics

Cognitively normal elderly participants (CN), patients with mild cognitive impairment due to AD (MCI-AD), and patients with mild dementia due to AD (MD-AD) did not differ according to education, depressive symptoms (Geriatric Depression Scale results), or Hachinski Ischemic Score (Kruskal–Wallis *p* > 0.05). In addition, none of the three groups differed according to sex (chi-square test, *p* > 0.05) (Table 1).

**Table 1:** Demographic and clinical characteristics of the participants.

	CN (N = 30)	MCI-AD (N = 30)	MD-AD (N = 30)
Male (%) *	13 (43.3%)	13 (43.3%)	12 (40.0%)
Years of education *	15 (3)	16 (2)	16 (3)
Age **	74 (7)	72 (10)	78 (4)
GDS*	5.5 (2)	5.5 (2)	5 (2)
HIS*	1 (1)	1 (1)	1 (0)

GDS, Geriatric depression scale; HIS, Hachinski Ischemic Score.

Data are represented as median and interquartile range unless specified otherwise.

*The groups did not differ significantly.

**MD-AD group differed significantly from CN and MCI groups. The CN and MCI groups did not show any significant differences.

The ages of participants in the MCI-AD and CN groups were similar (median age = 72 years, age range = 60–84 years for MCI-AD; median age = 74 years, age range = 63–89 years for CN). Participants in the MD-AD group were significantly older (median age = 78 years, age range = 65–85 years) (Kruskal–Wallis *p* < 0.05; post hoc analysis revealed significant differences between the CN and MD-AD groups; MCI-AD and MD-AD groups, and no significant difference between the CN and MCI-AD groups).

The demographic and clinical characteristics of the participants are presented in Table 1.

The results of the cognitive tests were significantly different among all three groups, as expected. Median MMSE score was 29 (range = 28–30) in CN, 26 (range = 24–27) in MCI-AD and 22 (range = 21–24) in MD-AD groups. Median ADAS-Cog score was 10.83 (range = 3.33–16.0) in CN, 20.84 (range = 12.67–33.0) in MCI-AD and 29.34 (range = 16.0–39.0) in MD-AD groups. Median CDR Sum of Boxes was 0 (range = 0–0) in CN, 2 (range = 1–3.5) in MCI-AD and 5 (range = 4–6) in MD-AD groups. Median fluency PAS score was 37 (range = 20–57) in CN, 28.5 (range = 14–56) in MCI-AD and 21 (range = 8–44) in MD-AD groups. Median fluency animals score was 20 (range = 11–36) in CN, 13 (range = 5–19) in MCI-AD and 10 (range = 3–19) in MD-AD groups. In all cases, Kruskal–Wallis *p* < 0.05, and post hoc analysis revealed significant differences among all three groups. The cognitive assessment results of the participants are presented in Table 2.

**Table 2:** Cognitive assessment results of the participants.

	CN (N = 30)	MCI-AD (N = 30)	MD-AD (N = 30)
MMSE *	29 (1)	26 (1)	22 (2)
ADAS-Cog 13 *	10.83 (4.58)	20.84 (4.42)	29.34 (5.66)
CDR Sum of Boxes *	0 (0)	2 (1)	5 (1)
Fluency PAS *	37 (13)	28.5 (9)	21 (13)
Fluency animals *	20 (7)	13 (6)	10 (5)

MMSE, Mini-mental state examination; ADAS-Cog 13, Alzheimer’s Disease Assessment Scale-Cognitive Subscale; CDR, Clinical Dementia Rating.

Data are represented as median and interquartile range.

*All three groups differed significantly.

### Odor identification

**Figure 1:**
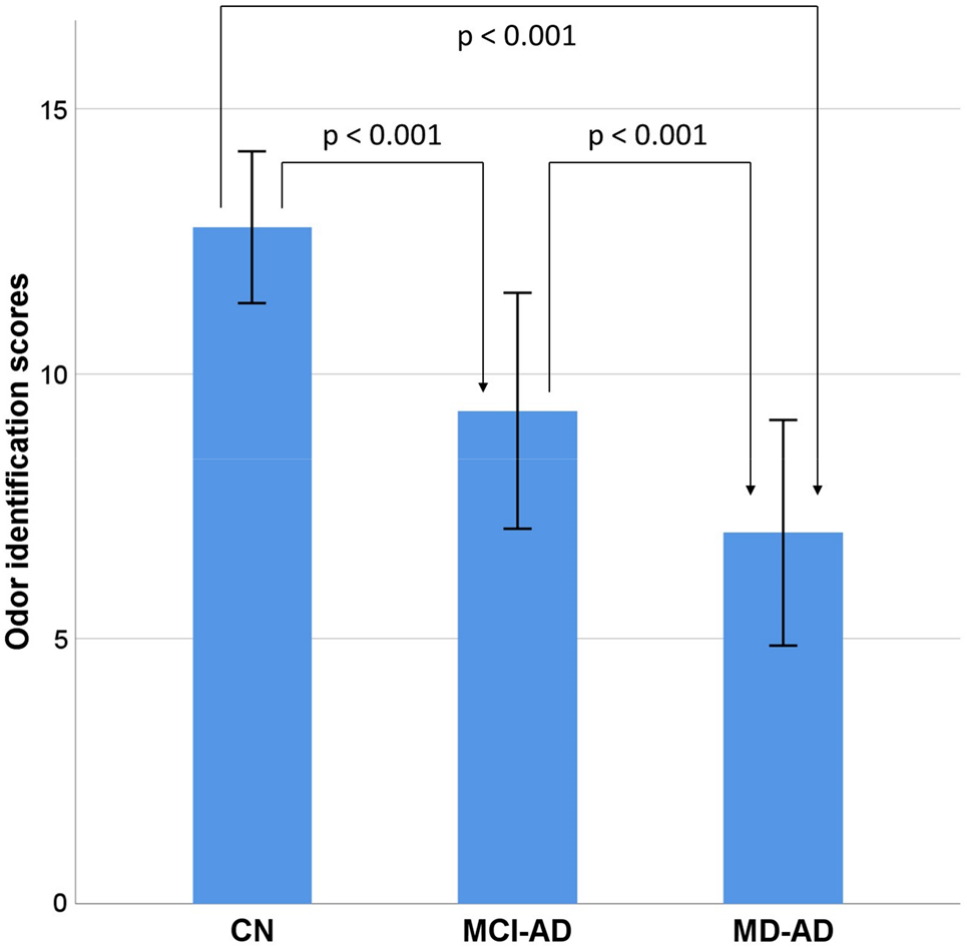
Odor identification scores in three groups of participants. Bars represent mean values, and error bars represent standard deviations.

One-way ANOVA revealed significant differences in odor identification scores among all three groups (mean and standard deviation: CN, 12.77 ± 1.43; MCI-AD, 9.3 ± 2.23; MD-AD, 7.0 ± 2.13; *p* < 0.001, the post-hoc analysis revealed significant differences among all three groups). The odor identification scores are presented in Fig. 1.

In the sample of all participants, odor identification scores were strongly correlated with the results of the cognitive tests: mini-mental state examination (MMSE) (Spearman’s rho: 0.735; *p* < 0.001), Alzheimer’s Disease Assessment Scale-Cognitive Subscale, version 13 (ADAS-Cog 13) (Spearman’s rho: − 0.771; *p* < 0.001), Clinical Dementia Rating (CDR) Sum of Boxes (Spearman’s rho: − 0.775; *p* < 0.001), and verbal fluency tests combined results (Spearman’s rho: 0.720; *p* < 0.001).

Upon analysis of the separate groups, no significant correlations were found between odor identification scores and cognitive test results in the MD-AD group. In the MCI-AD group, odor identification scores correlated with verbal fluency tests combined results (VFT) (Spearman’s rho: 0.627; *p* < 0.001). In the CN group, there were significant, although weak, correlations between odor identification scores and ADAS-Cog 13 scores (Spearman’s rho: –0.386; p = 0.035), as well as verbal fluency tests combined results (Spearman’s rho: 0.479; p = 0.007).

**Figure 2:**
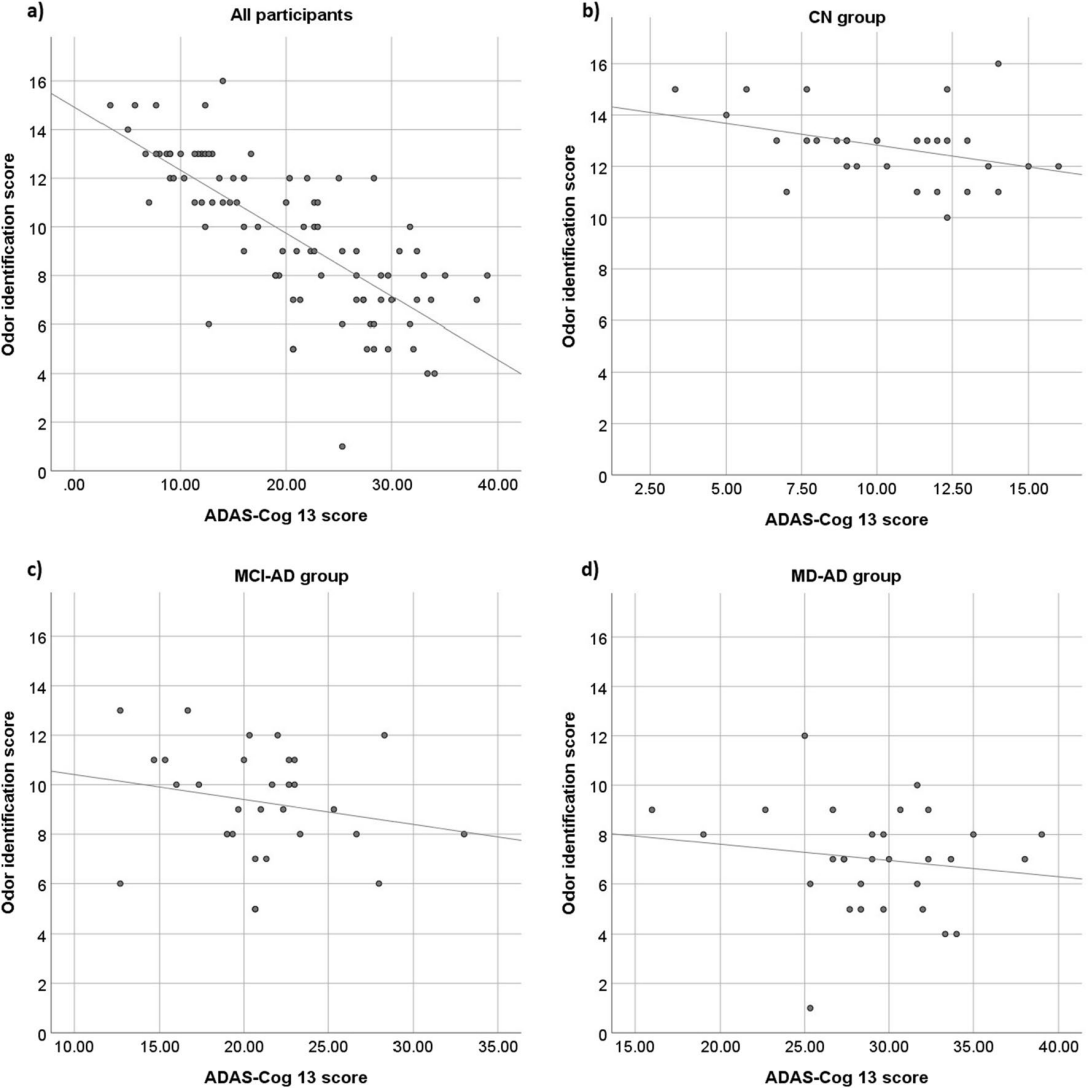
Relationship between odor identification scores and ADAS-Cog 13 results.

The relationship between odor identification scores and the ADAS-Cog 13 results is shown in Fig. 2. The relationships between odor identification scores and MMSE, CDR Sum of Boxes and VFT are shown in Supplementary Fig. S1, S2 and S3, respectively (online).

There was a significant, although weak, correlation between odor identification scores and age when the results of all participants were analyzed (Spearman’s rho: –0.334; p = 0.001). The correlation between odor identification and age remained significant in the CN group (Spearman’s rho: –0.365; p = 0.047) but not in the MCI-AD and MD-AD groups when analyzed separately. The relationship between odor identification scores and age is shown in Supplementary Fig. S4 (online).

Multiple linear regression models with age, sex, education, and cognitive test scores (MMSE, ADAS-Cog-13, CDR Sum of Boxes, and composite verbal fluency test score [VFT = fluency PAS + fluency animals]) as independent variables were tested to determine whether they significantly predicted odor identification scores.

The overall regression was statistically significant in all four models: model with MMSE (R^2^ = 0.525, F = 23.452, *p* < 0.001), model with ADAS-Cog 13 (R^2^ = 0.560, F = 27.079, *p* < 0.001), model with CDR Sum of Boxes (R^2^ = 0.569, F = 28.068, *p* < 0.001), and model with VFT (R^2^ = 0.464, F = 18.403, *p* < 0.001).

However, only cognitive test scores significantly predicted odor identification scores in each case (MMSE: β = 0.702, *p* < 0.001; ADAS-Cog 13: β = –0.735, *p* < 0.001; CDR Sum of Boxes: β = –0.735, *p* < 0.001; VFT: β = 0.719; *p* < 0.001). None of the other predictors (age, sex, and education) significantly predicted odor identification scores in any of the models (*p* > 0.05).

### Diagnostic characteristics of odor identification

Receiver operating characteristic (ROC) curve analysis was performed to evaluate the performance of the odor identification score in differentiating the CN group from AD (MCI-AD or MD-AD), MCI-AD, and MD-AD patients and MCI-AD patients from MD-AD patients. The ROC curves with areas under the curve (AUC) are shown in Supplementary Fig. S5 (online).

A cut-off score of ≤ 11 correct responses indicating AD was chosen.

Using this cut-off score for differentiating AD patients (MCI-AD or MD-AD) from CN participants, odor identification had sensitivity and specificity of 90% (95% CI: 79.49–96.24%) and 80% (95% CI: 61.43–92.29%), respectively. In addition, the negative and positive predictive values were 80% (95% CI: 64.71–89.72%) and 90% (95% CI: 81.41–94.87%), respectively. The overall diagnostic accuracy was 86.67% (95% CI: 77.87–92.92%).

The diagnostic characteristics remained good when differentiating MCI-AD patients from CN participants. Using the same cut-off score of ≤ 11, odor identification had a sensitivity and specificity of 83.33% (95% CI 65.28–94.36%) and 80% (95% CI: 61.43–92.29%), respectively. In addition, the negative and positive predictive values were 82.76% (95% CI: 67.89–91.59%) and 80.65% (95% CI: 66.68–89.66%), respectively. The overall diagnostic accuracy was 81.67% (95% CI: 69.56–90.48%).

**Figure 3:**
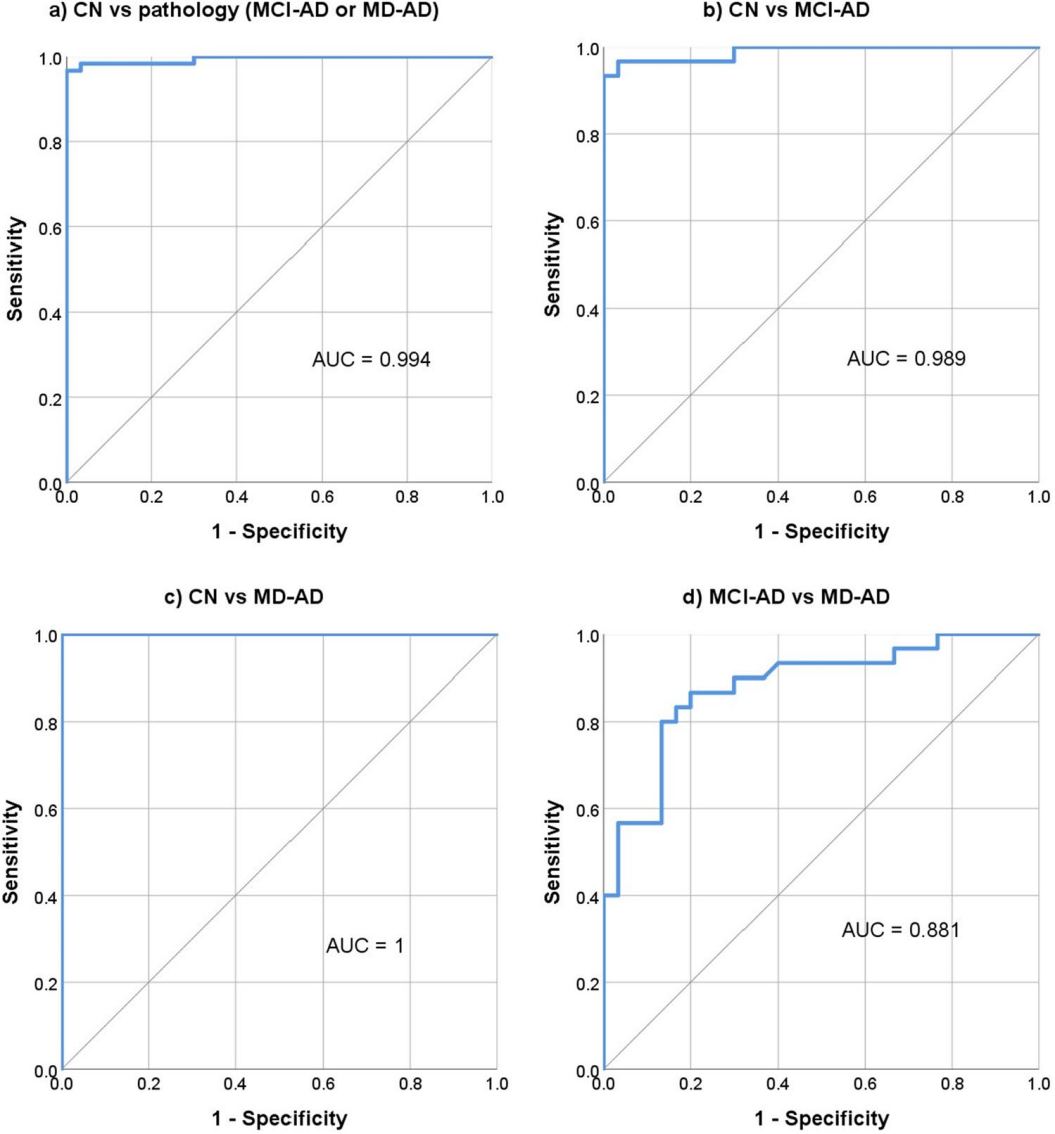
Performance of odor identification to ADAS-Cog 13 score ratio in differentiating between groups of participants.

The ratio of odor identification scores to ADAS-Cog 13 scores was calculated. Receiver operating characteristic (ROC) curve analysis was performed to evaluate the performance of this ratio in differentiating the CN group from AD (MCI-AD or MD-AD), MCI-AD, and MD-AD patients and MCI-AD patients from MD-AD patients. The ROC curves with areas under the curve (AUC) are shown in Fig. 3.

A cut-off score of ≤ 0.75 indicating AD was chosen.

Using this cut-off score for differentiating AD patients (MCI-AD or MD-AD) from CN participants, ratio of odor identification score to ADAS-Cog 13 score had sensitivity and specificity of 96.67% (95% CI: 88.47 – 99.59%) and 96.67% (95% CI: 82.78– 99.92%), respectively. In addition, the negative and positive predictive values were 93.55% (95% CI: 78.75–98.27%) and 98.31% (95% CI: 89.41–99.75%), respectively. The overall diagnostic accuracy was 96.67% (95% CI: 90.57–99.31%).

The diagnostic characteristics remained good when differentiating MCI-AD patients from CN participants. Using the same cut-off score of ≤ 0.75, ratio of odor identification score to ADAS-Cog 13 score had a sensitivity and specificity of 93.33% (95% CI 77.93–99.18%) and 96.67% (95% CI: 82.78–99.92%), respectively. In addition, the negative and positive predictive values were 93.55% (95% CI: 79.14–98.23%) and 96.55% (95% CI: 80.26–99.48%), respectively. The overall diagnostic accuracy was 95% (95% CI: 86.08–98.96%).

Multinomial logistic regression was performed to analyze the relationship between predictor variables and membership in the three groups (CN, MCI-AD, and MD-AD).

First, a model using age, education, sex, and ADAS-Cog 13 scores as predictor variables was tested. The fit between the model containing only the intercept and the data improved with the addition of predictor variables (X^2^ = 139.656, *p* < 0.001; Nagelkerke R^2^ = 0.887). Pearson’s X^2^ and Deviance X^2^ tests indicated that the model exhibited a good fit for the data (*p* > 0.05). The overall percentage of correctly classified cases using this model was 82.2% (93.3% CN, 70% MCI-AD, and 83.3% MD-AD cases correctly classified), with the ADAS-Cog 13 score as the strongest and most significant predictor (X^2^ = 122.652, *p* < 0.001).

In the second model, ADAS-Cog 13 scores were replaced with the ratio of odor identification score to ADAS-Cog 13 score. The model with age, education, sex, and the ratio of odor identification score to ADAS-Cog 13 score as predictor variables also showed a significant improvement in fit over a null model (X^2^ = 139.767, *p* < 0.001; Nagelkerke R^2^ = 0.887). Pearson’s X^2^ and Deviance X^2^ tests indicated that the model exhibited a good fit for the data (*p* > 0.05). The overall percentage of correctly classified cases using this model was 87.8% (96.7% CN, 83.3% MCI-AD, and 83.3% MD-AD cases correctly classified), with the ratio of odor identification score to ADAS-Cog 13 score as the strongest and most significant predictor (X^2^ = 122.763; *p* < 0.001).

## Discussion

In the current study, we found that odor identification was significantly impaired in the prodromal stage of AD (MCI-AD patients), and the impairment was even more severe in the later stages of the disease (mild dementia patients). This confirms the findings of previous studies, where olfactory impairment was also found to be present in the earliest stages of AD and further worsened during disease progression^10,14^.

We compared our results with normative data and results from previous studies on AD patients, in which the Sniffin’ Sticks odor identification test was used. The performance of healthy elderly participants varies from 12.06 ± 2.31 to 13.0 ± 0,92 according to normative data^30,31^. In addition, the performance of patients with MCI varied from 9.3 ± 4.0 to 10.2 ± 2.5 and that of patients with AD varied from 6.7 ± 2.3 to 7.8 ± 3.4 in various studies^14,32,33^. Therefore, our results were within the range of previous results and normative data. Collectively, the results from the current study and previous research suggest that impairment of odor identification is fairly consistent across various samples of patients with AD. Therefore, odor identification testing can be applied to various populations. Interestingly, odor identification is influenced by a patient’s personal and cultural experiences and familiarity with different odors^34^. This makes it necessary to adapt odor identification tests to different populations^35–37^. However, when comparing results from studies conducted in Germany, China, the United States of America, and Lithuania (the current study), the results were rather similar, even though all of them used the standard Sniffin’ sticks odor identification set^14,32,33^.

Odor identification scores were strongly and significantly correlated with the results of the cognitive assessment. Linear regression analysis further demonstrated a significant relationship between these variables. However, age, sex, and education did not significantly predict odor identification scores in the linear regression models. These findings are in accordance with structural and functional changes in the olfactory system, which have been demonstrated in previous studies, and further prove that olfactory impairment is associated with processes of AD itself and cannot be explained by other factors that have been proven to influence olfaction in the general population, such as age and sex^24–29^. As olfactory and memory systems are known to significantly overlap anatomically and both are affected by cholinergic deficit, present in AD, the relationship between olfactory and cognitive impairment might be related pathogenetically. However, further studies are needed on this subject.

Odor identification demonstrated excellent characteristics for the differentiation of AD patients from healthy controls (AUC = 0.949) and for the differentiation of prodromal AD (MCI-AD) patients from healthy controls (AUC = 0.908). However, the results were not as good when differentiating between the different stages of AD (MCI-AD vs. MD-AD, AUC = 0.773). Similar results were found in previous studies, where odor identification also had better qualities in differentiating healthy participants from patients with AD than in differentiating between different stages of AD^33^. This suggests that changes in odor identification occur early in the course of the disease and are pronounced even in the prodromal stage of AD, thus making odor identification testing very suitable for screening for early AD and less reliable for monitoring disease progression.

However, diagnosing early AD is the most challenging task in clinical practice, especially in primary care settings. More than half (51%) of primary care physicians surveyed by the Alzheimer's Association said they were uncomfortable diagnosing MCI due to AD^38^. Currently available biomarkers are not able to solve this issue, as they are not easily accessible in community settings. Lack of specialists and facilities to perform diagnostic testing was the most commonly cited challenge by primary care physicians in the United States of America, when diagnosing mild cognitive impairment (MCI) due to AD^38^. Objective olfactory testing may be very useful for improving diagnostic certainty of screening for early-stage AD. In particular, 72% of primary care physicians stated that they find it challenging to differentiate MCI from normal aging^38^. As odor identification proved to have excellent characteristics in differentiating MCI-AD patients from healthy controls, it could be very helpful with this task.

The ratio of odor identification score to ADAS-Cog 13 score had even better characteristics for the differentiation of AD patients from healthy controls (AUC = 0.994) and for the differentiation of prodromal AD (MCI-AD) patients from healthy controls (AUC = 0.989). Using a combined measure of odor and cognitive testing would be very useful, as that could help to not only improve the detection of early-stage AD patients, but also differentiate them from patients with other disorders known to affect olfaction, such as Parkinson’s disease^39^. However, further studies are needed to confirm that.

Substitution of ADAS-Cog 13 score with a ratio of odor identification score to ADAS-Cog 13 score in a multinomial logistic regression model containing demographic and cognitive data as predictive variables improved the overall classification accuracy from 82.2% to 87.8%. Correct classification of MCI-AD cases improved the most (from 70% to 83.3%), once again confirming the additional value of odor identification testing in screening for AD at the early stages.

The present study has several limitations. First, CSF and PET biomarkers were not used in this study. Studies involving these factors would help to explore the relationship between olfactory impairment and brain amyloid-beta (Aβ) deposition and neuronal degeneration markers. Also, CSF and PET biomarkers would help in excluding mild cognitive impairment due to dementia with Lewy bodies, which is difficult to differentiate from MCI due to AD with cognitive and olfactory testing alone, according to studies^40^. No participants in the current study had evidence of Parkinsonism, prominent visual hallucinations, or rapid eye movement sleep abnormalities, as required by criteria for probable AD and MCI due to AD by NIA-AA^4,5^. However, CSF and PET biomarkers would help in confirming the diagnosis. Second, the sample size was sufficient to prove significant changes; however, studies involving larger sample sizes would be useful in confirming these findings. Finally, the cross-sectional design of the study did not allow precise conclusions to be drawn regarding the progression of changes during the course of AD. Even though the results of the current study are encouraging, longitudinal studies are needed in order to confirm the value of odor identification testing in screening for AD.

## Conclusion

In conclusion, our findings indicate that odor identification is impaired in the prodromal stage of AD and that these changes progress during the course of the disease. Odor identification testing demonstrates good diagnostic qualities and can improve the accuracy of screening for early-stage AD, serving as a reliable, noninvasive, and affordable marker.

## Methods

### Participants

Ninety participants were enrolled in the study: 30 CN participants, 30 patients with MCI-AD, and 30 patients with MD-AD. Cognitively normal participants had no cognitive complaints and a CDR score of 0. MCI-AD patients met the National Institute on Aging-Alzheimer’s Association (NIA/AA) criteria for MCI-AD (Albert et al., 2011^4^) and had a CDR score of 0.5. Furthermore, patients with MD-AD met NIA/AA criteria for probable AD (McKhann et al., 2011^5^) and had a CDR score of 1.

Participants were excluded from the study if they had any central nervous system (CNS) disorder other than MCI-AD or MD-AD. Other exclusion criteria based on possible effects on cognitive functioning were cerebrovascular disorders (Hachinski Ischemic Score ≥ 4), severe head trauma, psychosis, depression (Geriatric Depression Scale > 9), psychoactive medications, and substance abuse. Participants were also excluded from the study if they had conditions potentially affecting olfactory function, such as nasal surgery, significant exposure to volatile substances, recent viral infections, or smoking.

The study was approved by the Vilnius Regional Bioethics Committee (approval number 2021/6–1355-830), and all the experiments were performed in accordance with the Declaration of Helsinki. In addition, written informed consent was obtained from all participants before the study.

### Assessments of cognitive function

Cognitive and functional performance was evaluated using the CDR scale. MMSE was performed to evaluate global cognition. A more detailed evaluation of cognitive functioning was performed using the ADAS-Cog (scores ranging from 0–70) with additional delayed recall and number cancellation tasks (ADAS-Cog 13, scores ranging from 0–85). The delayed recall was scored from 0–10 (the number of words not recalled). The number cancellation task was scored from 0 to 5 (0 representing the best [≥ 30 correct responses] and 5 representing the worst [0–5 correct responses] performance). Verbal fluency was also tested (PAS and animal naming tasks).

### Assessment of odor identification

The Sniffin’ Sticks odor identification test was performed (Burghart®, Wedel, Germany). The Sniffin’ Sticks odor identification test consists of 16 odors presented in felt-tip pens. The odors are orange, leather, cinnamon, peppermint, banana, lemon, liquorice, turpentine, garlic, coffee, apple, clove, pineapple, rose, anise and fish. Each odor was presented only once, for 3–4 s. The time interval between odors was 30 s. Participants were asked to select one of the four items from the answering card that best described the odor even if they were uncertain. The odor identification score is the number of correct responses out of sixteen. The examiner used odorless gloves, and the participants were instructed not to eat or drink anything at least 15 min prior to testing, as per the test instructions.

### Data analysis

Statistical analysis was performed using IBM SPSS Statistics version 26.0. The normality of data distribution was tested using the Shapiro–Wilk test. A two-tailed chi-square test (for categorical variables) and Kruskal–Wallis or one-way analysis of variance (ANOVA) tests (for numerical variables) were used to analyze differences between groups. The correlations between variables were analyzed using Spearman rank correlation coefficients. Linear regression models were created to analyze the predictions of continuous variables. Categorical variables were predicted using multinomial logistic regression. ROC curve analysis was performed to evaluate the accuracy of the diagnostic tests. Statistical significance was set at a p-value of < 0.05.

## Supplementary Information

Supplementary Information.

## Data Availability

The datasets used and analyzed during the current study are available from the corresponding author upon reasonable request.
