# Supplementary material for: Properties of odor identification testing in screening for early-stage Alzheimer’s disease
Source: Sci Rep. 2023 Apr 13;13:6075. doi: 10.1038/s41598-023-32878-w (PMC10102162; doi:10.1038/s41598-023-32878-w)

**Fig. S1.** Relationship between odor identification scores and MMSE results


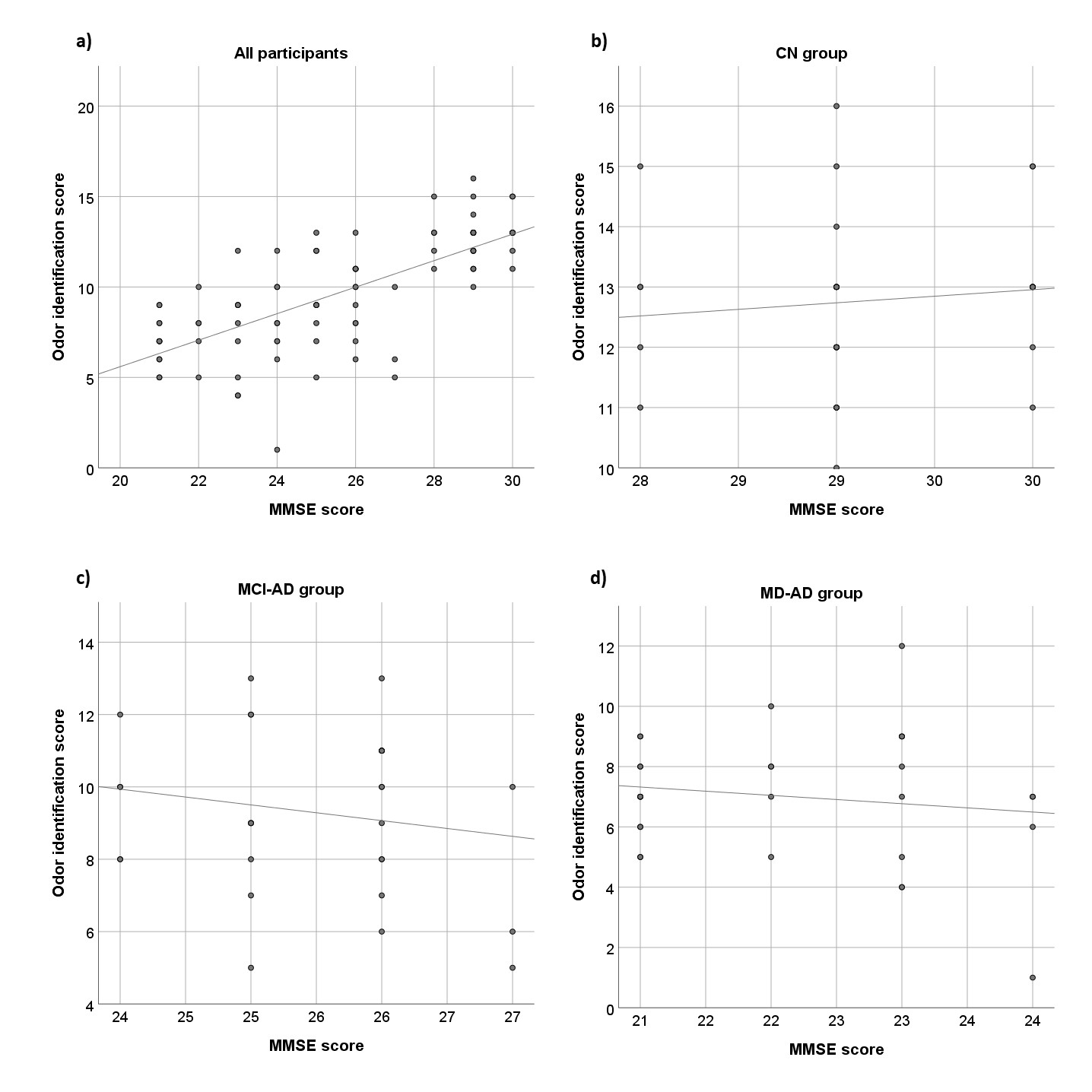


**Fig. S2.** Relationship between odor identification scores and CDR Sum of Boxes


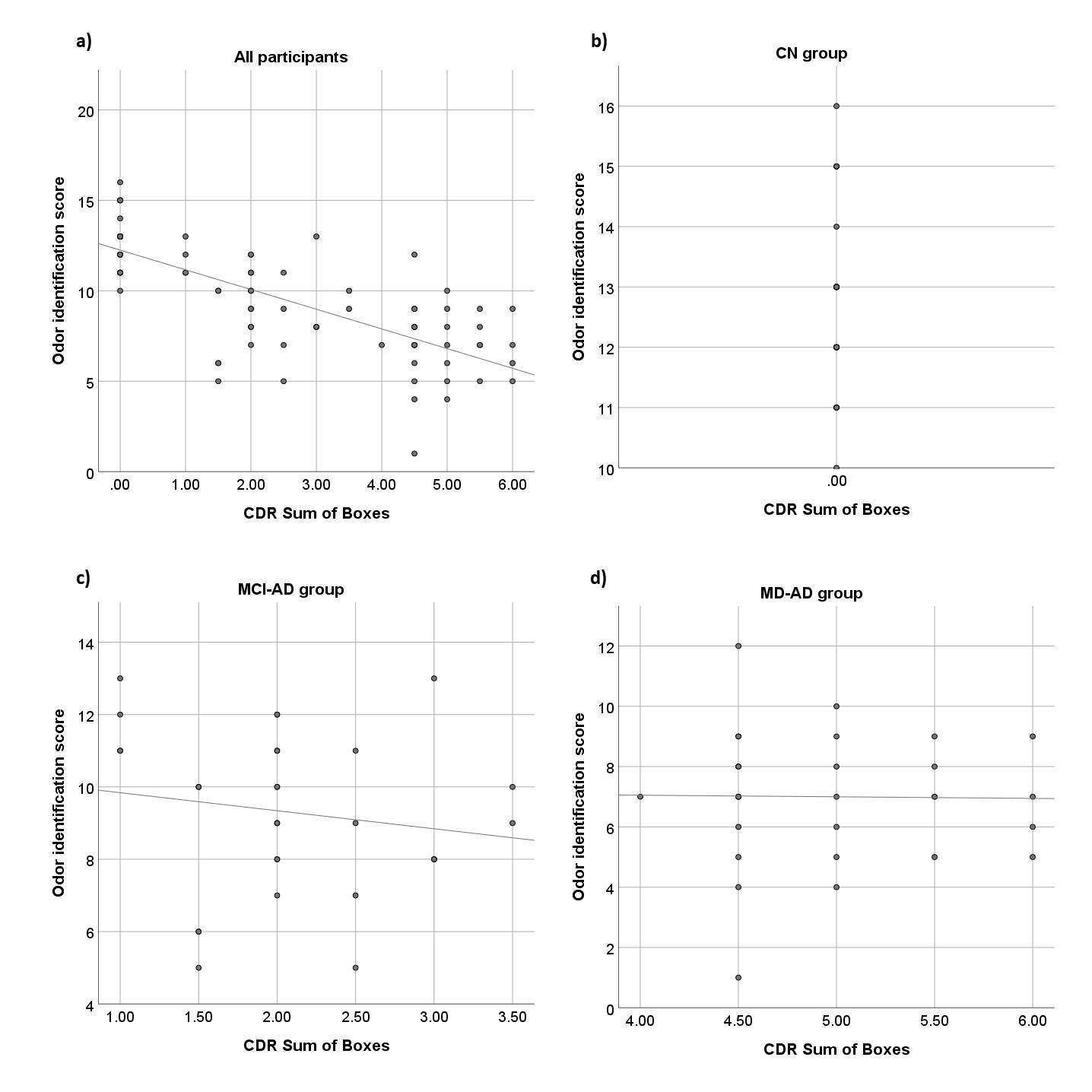


**Fig. S3.** Relationship between odor identification scores and VFT results


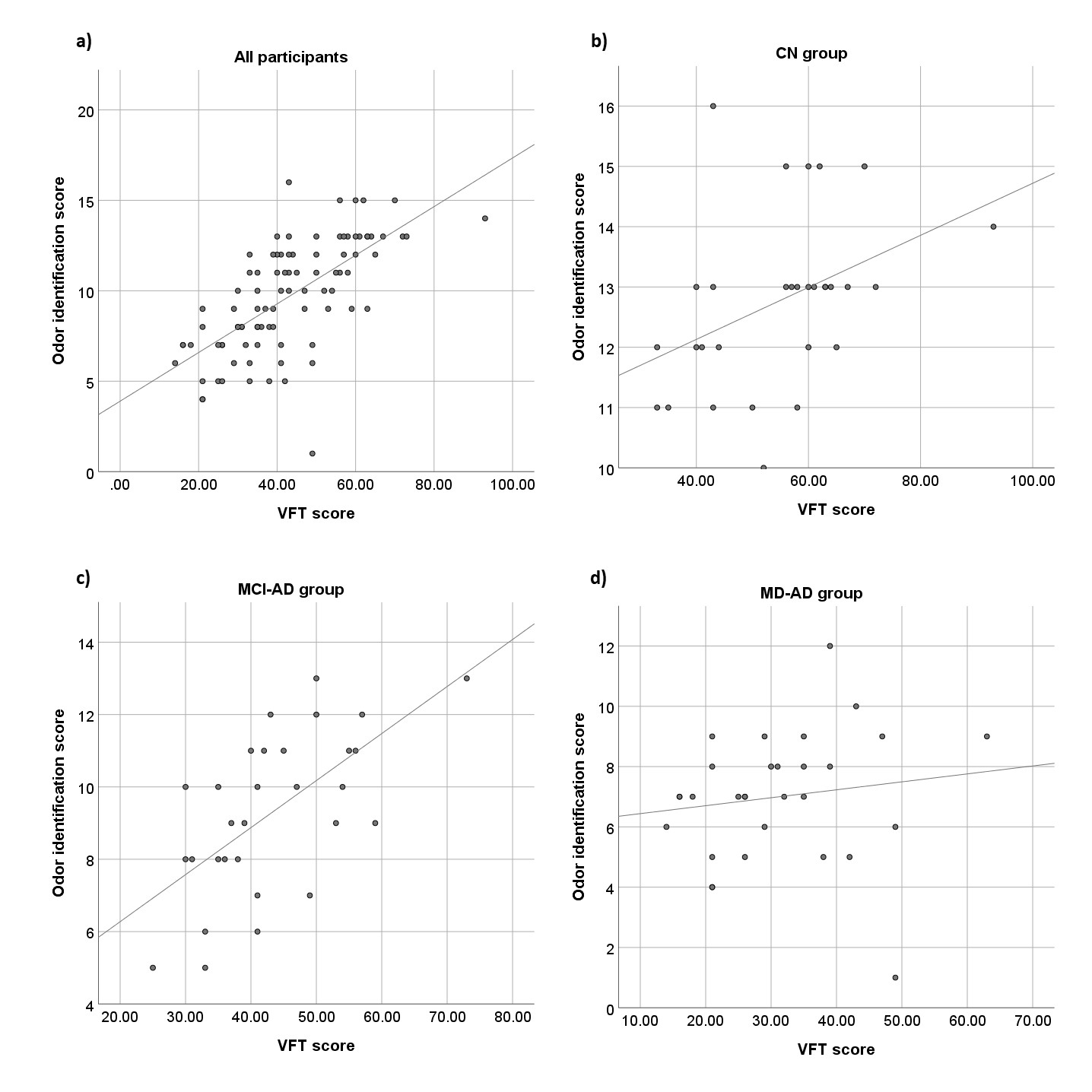


**Fig. S4.** Relationship between odor identification scores and age


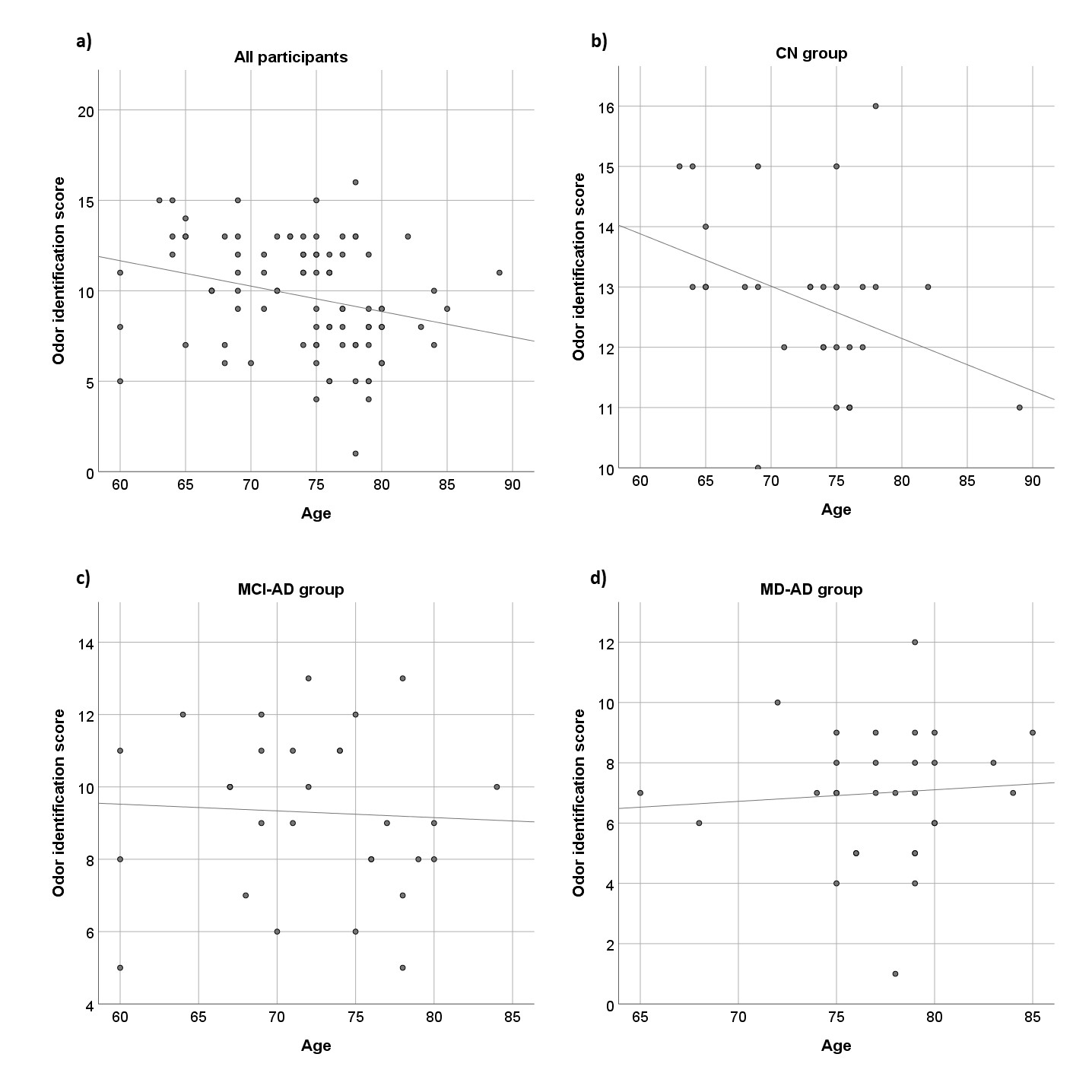


**Fig. S5.** Performance of odor identification score in differentiating between groups of participants


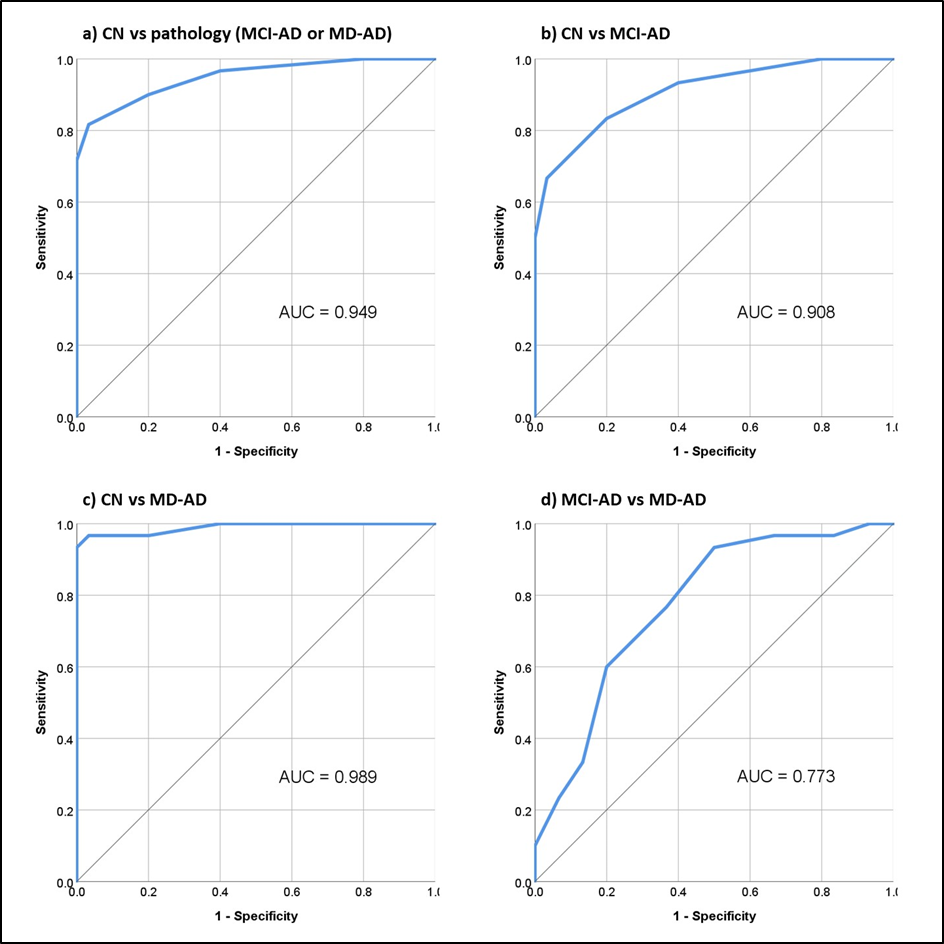

Supplement: Supplementary file 1 — Supplementary Information. [file 41598_2023_32878_MOESM1_ESM.docx]
